# Applications of Pristine and Functionalized Carbon Nanotubes, Graphene, and Graphene Nanoribbons in Biomedicine

**DOI:** 10.3390/nano11113020

**Published:** 2021-11-10

**Authors:** Maria G. Burdanova, Marianna V. Kharlamova, Christian Kramberger, Maxim P. Nikitin

**Affiliations:** 1 Center for Photonics and 2D Materials, Moscow Institute of Physics and Technology, Institutskii Pereulok 9, 141700 Dolgoprudny, Russia; burdanova.mg@mipt.ru; 2 Department of Physics, Moscow Region State University, Very Voloshinoy Street, 24, 141014 Mytishi, Russia; 3 Phystech School of Biological and Medical Physics, Moscow Institute of Physics and Technology, Institutskii Pereulok 9, 141700 Dolgoprudny, Russia; max.nikitin@phystech.edu; 4 Institute of Materials Chemistry, Vienna University of Technology, Getreidemarkt 9/BC/2, 1060 Vienna, Austria; 5 Faculty of Physics, University of Vienna, Strudlhofgasse 4, 1090 Vienna, Austria; christian.kramberger-kaplan@univie.ac.at

**Keywords:** carbon nanotubes, graphene, graphene nanoribbons, functionalization, biosensing, bioimaging, drug delivery, biological applications, nanomaterial toxicity

## Abstract

This review is dedicated to a comprehensive description of the latest achievements in the chemical functionalization routes and applications of carbon nanomaterials (CNMs), such as carbon nanotubes, graphene, and graphene nanoribbons. The review starts from the description of noncovalent and covalent exohedral modification approaches, as well as an endohedral functionalization method. After that, the methods to improve the functionalities of CNMs are highlighted. These methods include the functionalization for improving the hydrophilicity, biocompatibility, blood circulation time and tumor accumulation, and the cellular uptake and selectivity. The main part of this review includes the description of the applications of functionalized CNMs in bioimaging, drug delivery, and biosensors. Then, the toxicity studies of CNMs are highlighted. Finally, the further directions of the development of the field are presented.

## 1. Introduction

Carbon nanomaterials (CNMs), such as graphene, graphene oxide (GO), graphene quantum dots (GQDs), graphene nanoribbons (GNRs), and carbon nanotubes (CNTs), are of interest to the research community due to their outstanding chemical, optical, electrical, and mechanical properties [1,2,3,4]. They combine ultra-lightweight characteristics with mechanical strength, flexibility, as well as excellent electrical and thermal conductivity [5,6,7,8,9]. Their high specific surface area provides the means to load and deliver diagnostic and therapeutic agents to tissues and organs. CNMs are not only able to cross the cellular membrane, but are also viable probes for spectroscopic diagnostics by resonantly enhanced Raman scattering, strong nearinfrared optical absorption, high photoluminescence yield, and photoacoustic response [10]. These properties can be utilized in a wide range of biomedical applications, involving targeted drug delivery and anticancer therapy, which are monitored by bioimaging.

Conventional chemotherapeutic drugs have often limited solubility in biologic media, low stability, and relatively high toxicity for normal cells and tissues and are missing a selective biodistribution. These shortcomings can be overcome if CNMs are used as targeted drug carrier and delivery systems [11]. While simple pure CNMs also pose significant issues such as toxicity, low solubility, a nonbiodegradable nature, and the formation of agglomerates, they do provide a platform for chemical surface functionalization, which can specifically address these issues [12,13]. The emergence of new techniques for surface functionalization is crucial to reduce or even eliminate the disadvantageous properties of pristine CNMs. For instance, the chemical surface functionalization of CNMs can greatly improve their solubility in watery solutions. Solubility is a prerequisite for excretability and general biocompatibility under physiological conditions [14]. The effects of the surface functionalization of CNMs can be accessed via the blood circulation time. Chemical surface functionalization establishes functional groups that are available for further bioconjugation with therapeutic and targeting agents. Targeting agents can specifically discriminate between healthy and diseased tissue, resulting in smart delivery systems [13,15,16,17]. The surface functionalization of CNMs can also improve their cellular uptake, leading to a well-focused therapeutic effect.

The aim of this review is to comprehensively describe the latest achievements in the chemical functionalization routes and biological applications of CNTs, graphene, and its derivatives, such as GNRs, in biomedicine. The review has the following structure. It starts from the description of noncovalent and covalent exohedral modification approaches, as well as endohedral functionalization methods. After that, the improvement of the functionalities of CNMs is highlighted. These methods include the functionalization to improve hydrophilicity, biocompatibility, blood circulation time and tumor accumulation, and the cellular uptake and selectivity. Then, the applications of functionalized CNMs in bioimaging, drug delivery, and biosensors are reviewed. Finally, the toxicity studies of CNMs are highlighted.

## 2. Overview of Carbon Nanomaterials for Biomedical Applications

CNTs are hollow cylindrical molecules that consist of one or several rolled-up sheets of hexagonal networks (graphene) with at least one end typically capped with a hemisphere of buckyballs. The distinct property of CNTs is their high aspect ratio: their diameter is about one nanometer, while their length can exceed tens of micrometers. Unlike other nanomaterials, they can be metallic or semiconducting, depending on their chirality and add-atoms. This results in unique electronic, mechanical, and optical properties, ideal for a wide range of purposes [18]. In addition, CNTs have a small diameter, high curvature, and large surface area, which allow them to effectively interact with biomolecules through van der Waals forces, via π–π stacking, and hydrophobic interactions [19]. These properties can also be used to facilitate the surface modification of CNTs to increase their solubility in aqueous media or modulate the covalent attachment of functional groups for biomedical applications.

Graphene is a single layer of a honeycomb carbon lattice, the basic building block of all graphitic forms. Graphene and its oxidized derivatives (GO or reduced graphene oxide (rGO)) uniquely combine high electronic and thermal conductivities, mechanical strength, and impermeability to gases [20]. Graphene interacts with biomolecules through π–π stacking and/or electrostatic interactions, which are of great value for drug loading and biosensor design applications [21]. In addition, the rich oxygen-containing functional groups that are attached to graphene oxide (GO) can be directly functionalized by biological ligands to facilitate targeted imaging and drug delivery. While graphene could be cytotoxic, pharmaceutical and biomedical applications can benefit greatly from nontoxic, biocompatible, and water-dispersible graphene layers that are produced through chemical functionalization with various ligands.

Another recently invented and attractive biomaterial from the carbon family is GNRs, which is defined as a strip of graphene with a width less than 100 nm. GNRs are remarkable materials due to their attractive chemical, electrical, and optical properties [22]. In comparison to a number of nanomaterials, GNRs exhibit superior biocompatibility and resistance to photobleaching. In addition, GNRs’ semiconducting property can be switched to semimetal by changing its width [23]. This nanomaterial carries keen features of graphene, such as a large surface area and available π electrons, which make it a smart material for a wide range of biomedical applications [24].

## 3. Advantages and Disadvantages of Carbon Nanomaterial

Each type of CNMs has advantages, disadvantages, and unique properties due to their distinctive characteristics. At the same time, CNTs, graphene, and GNRs share properties of an extremely small size and light weight, making them ideal candidates for nanoapplications. They all are resistant to ambient conditions especially to temperature changes from extreme cold to extreme heat and have unique mechanical properties. Moreover, they are not biodegradable. CNMs have large specific surface areas on which a wide range of chemicals can be attached. Therefore, CNMs can be functionalized to enhance their physicochemical properties and to deliver molecules and even large particles. In addition, CNMs are highly conducting in nature, provided they have a particular structure. In comparison to graphene, CNT and GNRs have optical properties, for example bright photoluminescence (PL), which are highly related to their geometrical structure.

At the moment, the process is relatively expensive to produce CNMs, and in most cases, only small quantities of the material can be produced. Despite the advantages of using CNMs, it would be difficult to replace the older well-established technology with a new one. The lack of solubility in aqueous media, as well as their toxicity caused by the hydrophobic surface make CNMs difficult for some specific applications. An additional factor that will affect CNMs’ applications is the ability to form large colloidal aggregates without functionalization. CNMs cannot survive from oxygen along with heat. Furthermore, their properties are highly dependent on their size, which in some cases is difficult to control. Scaling up CNMs to useful sizes has not produced materials with the same properties.

## 4. Types of Chemical Functionalization of Carbon Nanomaterials

**Figure 1:**
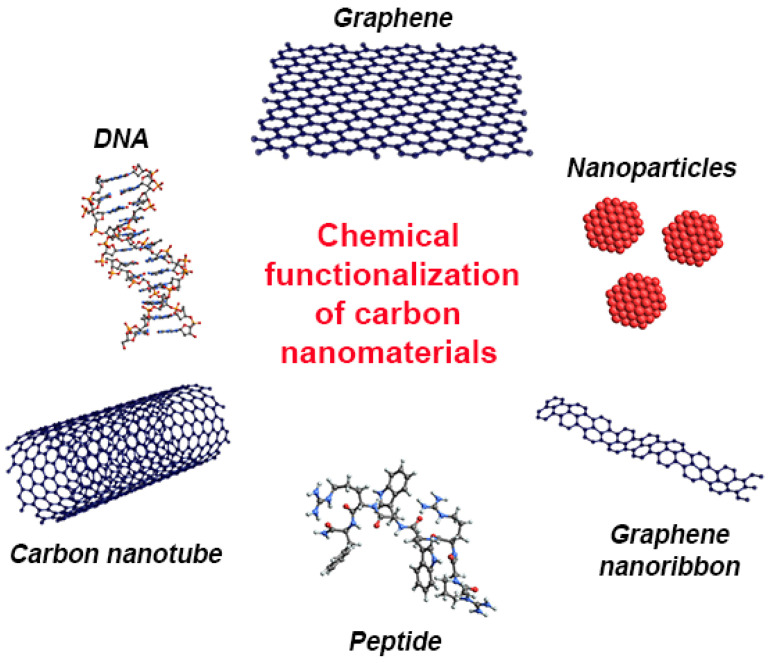
A schematic representation of different types of chemical functionalization of CNMs.

Chemical functionalization of CNMs is performed by two methods: exohedral and endohedral functionalization. There are two techniques for the surface functionalization of CNMs: noncovalent and covalent functionalization. In the next sections, we provide an overview of the chemical functionalization approaches of CNMs (Figure 1).

### 4.1. Exohedral Chemical Functionalization

#### 4.1.1. Noncovalent Chemical Functionalization

Noncovalent functionalization relies on other, generally weaker, but also reversible interactions. These can be hydrophobic interactions, π–π interactions, and/or van der Waals forces between CNMs and guest molecules. CNMs can be wrapped by many amphiphilic molecules, including pyrene, naphthalene derivatives, proteins, ribonucleic acid (RNA), deoxyribonucleic acid (DNA), peptides, polymers, and surfactants that are readily adsorbed onto CNMs by noncovalent interactions. The absorbed molecules can be the bonding sites for further functionalization with therapeutic and targeting agents. There is a substantial amount of literature dedicated to the noncovalent chemical modification of CNMs. The reader can refer to several reviews [25,26].

#### 4.1.2. Covalent Chemical Functionalization

Covalent functionalization is the covalent bonding of desired functional groups on the surface of CNMs. The stable bonds enable a better process control as compared to noncovalent functionalization. The covalent bond distorts the conjugated π electron framework of CNMs by locally switching from sp${}^{2}$ to sp${}^{3}$ hybridization, thereby introducing defects in the honeycomb carbon lattice. Surface oxidation, cycloaddition reactions, and radical additions are common techniques to start the covalent functionalization of CNMs. The primary covalent groups can then be used for subsequent derivatizations with biologically relevant molecules.

Refluxing and sonication in oxygen-containing mineral acids and applying ozone or hydrogen peroxide are established methods for the oxidative treatment of CNMs. The oxygen-containing groups that are created by the initial oxidative treatment become the anchor sites for esterification or amidation reactions. These are widely employed for the conjugation of water-soluble organic molecules, hydrophilic polymers such as polyethylene glycol, nucleic acids, or peptides. These steps yield a wide variety of multifunctional CNMs. Another common approach is cycloaddition reactions by 1,3-dipolar nitrene and carbene. Such functional groups can be substituted with amino acids, peptides, and fluorescent molecules. The covalent functionalization via radical addition reactions is conducted through aryl diazonium coupling. For more information, reader can refer to the reviews [26,27].

### 4.2. Endohedral Chemical Functionalization

If the chemical functionalization of CNTs is performed endohedrally, the functional molecules are encapsulated inside the channels of nanotubes [28,29,30,31,32,33,34,35,36,37,38,39,40,41]. This technique has two advantages. Firstly, the host CNTs are a physical barrier that protects the chosen diagnostic and therapeutic agents. They effectively block the oxidation or any other unwanted interactions in a chemical or biological environment. Secondly, the endohedral functionalization leaves the outer surface of CNTs available for exohedral functionalization with dispersing and targeting agents. In medical applications, specific antibodies, folic acid, or peptides provide the moieties to target and deliver the encapsulated diagnostic and therapeutic agents to specific cells.

The technique to achieve an endohedral functionalization or simply filling of CNTs has to be chosen according to the chemical ad physical stability of the respective filler molecules or substance [42,43,44,45,46,47,48,49,50]. High-temperature processes such as the capillary filling technique can be employed for salt melts such as metal halogenides or elemental metals. If the filler has a sufficient vapor pressure and thermal stability, gas phase filling can be employed [51,52,53,54,55,56,57,58,59,60,61,62,63]. Gas phase filling is of particular interest for filling with organic or organometallic precursors. Polycyclic aromatic precursors can then undergo polymerization inside the CNTs. Organometallic precursors can undergo a thermal decomposition to yield metal carbides or metals. If the intended filler is not suited for high temperatures, milder filling techniques have to be used. Supercritical CO${}_{2}$ extraction, nanoextraction, and nanocondensation are suited methods to insert biological and other heat-sensitive molecules into CNTs. The reader can refer to specialized reviews on the filling of CNTs [44,53,63,64].

## 5. Chemical Functionalization of Carbon Nanomaterials to Improve the Functionality in Biomedicine

**Figure 2:**
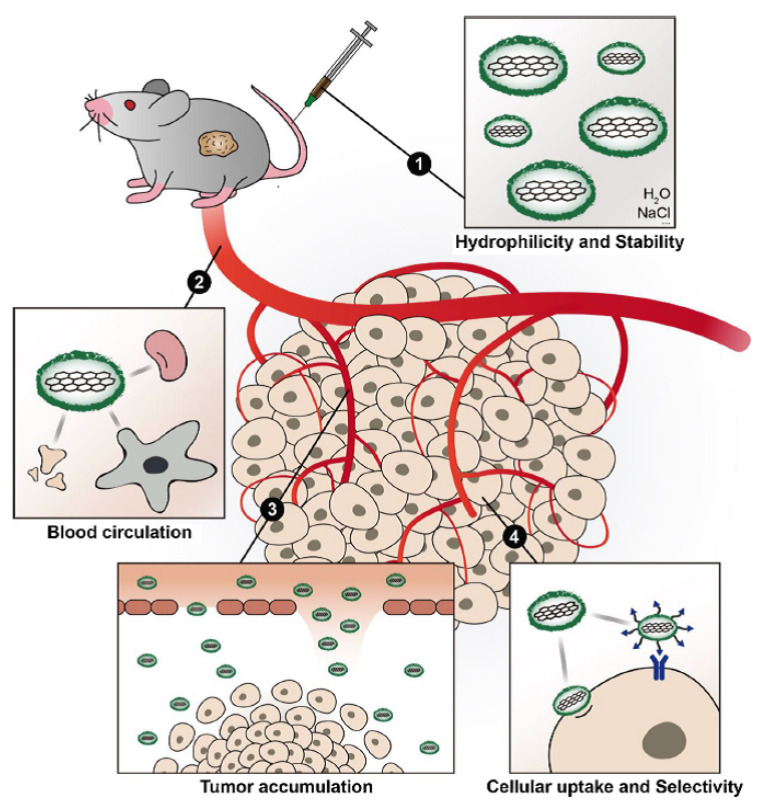
Main directions to improve the functionality of CNMs in biomedicine. Reprinted from [65]. Copyright 2018, with permission from Elsevier.

In the literature, there are reports on methods to improve the functionality of CNMs in biomedicine. These methods can be classified into four main directions, such as functionalization for (i) improving hydrophilicity, (ii) biocompatibility, (iii) the blood circulation time and tumor accumulation, and (iv) the cellular uptake and selectivity (Figure 2) [65].

### 5.1. Functionalization for Improving Hydrophilicity

CNMs should be soluble in aqueous solutions and preserve their physicochemical properties in the medium used for their administration and in biological fluids. To achieve hydrophilicity, CNMs such as GO are usually covalently functionalized with hydrophilic polymers containing amine groups through the formation of amide bonds.

Several studies [66,67] showed that GO was functionalized with amine-terminated polyethylene glycol (PEG). In [68,69], GO was functionalized with amine-terminated dextran. To achieve hydrophilicity, CNMs were also functionalized with chitosan [70], mesoporous silica [71], bovine serum albumin [72], octaarginine [73], glucose [74], and gelatine [75].

### 5.2. Functionalization for Improving the Biocompatibility

The biocompatibility of CNMs is influenced by the intrinsic properties of the materials, such as the surface chemistry, size, stability, purity, as well as by factors related to assay conditions, such as culture conditions, presence of serum, and cell line [76,77]. The cytotoxicity of CNMs is explained by the poor solubility and high toxicity of hydrazine hydrate, which is used in the majority of the reduction processes. To reduce the safety issues associated with the use of CNMs, they were functionalized with different types of hydrophilic and biocompatible materials.

Therefore, surface functionalization of graphene using biocompatible polymers to make it more water soluble and to improve its dispersibility are commonly used. In past works [78,79], GO was functionalized with branched amine-terminated PEG. In [80], BSA was used as modifier. For functionalization, the authors also used glutaraldehyde [81], L-cysteine [82], hematin-terminated dextran [83], polydopamine [84], dextran [85], chitosan [86], and heparin-terminated with dopamine [87]. In [88], nanographene sheets (NGSs) were firstly coated with polyethylene glycol and a fluorescent labeling agent and then secondly subjected to a study of their in vivo behaviors. In the biocompatibility study, mice were injected with PEGylated NGSs. Neither the histology, blood chemistry, nor complete blood panel analysis showed any obvious side effects. The authors of [89] investigated the long-term in vivo biodistribution and biocompatibility of ${}^{125}$I-labeled nanographene sheets functionalized with PEG. Mice were injected with a dose of 20 mg/kg and then monitored over three months by blood biochemistry, hematological analysis, and histological examinations. PEGylated NGSs did not show any appreciable level of toxicity over the whole period. In [78], the in vivo biodistribution and biocompatibility of pristine GO and a number of PEG-functionalized GO derivatives with different sizes and surface coatings were systematically investigated at high doses after oral and intraperitoneal administration. Histological examination of organ slices and hematological analysis revealed that the administered GO and PEGylated GO derivatives were retained in the mouse body over a long period of time. However, there was no apparent significant toxicity of the GO and PEGylated GO derivatives to the treated mice observed.

### 5.3. Functionalization for Improving the Blood Circulation Time and Tumor Accumulation

CNMs are not suited to overcome the biological barriers required to achieve a suitable tumor accumulation. Upon introduction to the blood stream, CNMs adsorb proteins and suffer internalization by macrophages [90]. The functionalization of CNMs can greatly reduce the interactions and thus improve the blood circulation time. Brunched amine-terminated PEG was also used to improve the blood circulation time and tumor accumulation of CNMs [11,91]. In [91,92], C18-phenylmethylene hydantoin (PMH)–PEG was used to achieve this aim.

### 5.4. Functionalization for Improving the Cellular Uptake and Selectivity

After reaching the tumor site, CNMs must be internalized by cancer cells. The functionalization of CNMs can improve the cellular uptake and selectivity. Methods of the efficient functionalization of CNMs have been developed. In several studies [66,93], branched amine-terminated PEG was used. For functionalization, the authors also employed carboxymethyl chitosan [94], amine-terminated dextran [69], glutaraldehyde [95], transferrin [66], polycyclic aromatic hydrocarbon (PAH) aldehyde [66], clorotoxin [96], polyvinylpyrrolidone (PVP) [97], hematin-terminated dextran [83], cholesteryl [98], and lactoferrin [99].

## 6. Application of Functionalized Carbon Nanomaterials in Biomedicine

**Figure 3:**
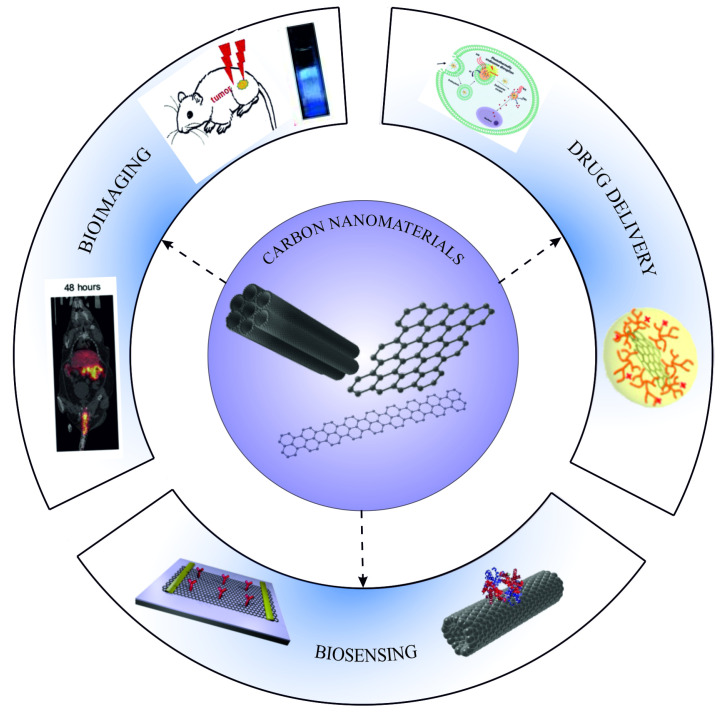
The primary application of graphene-based nanomaterials in biomedicine: bioimaging, drug delivery, biosensing. Some of the included images were adapted with permission from [104,105]. Copyright 2013, American Chemical Society. Copyright 2016, with permission from Elsevier.

Chemically functionalized CNMs can be used for bioimaging and in vivo monitoring of processes in living cells, tissues, or even whole bodies. Such tools are relevant for research and diagnostics alike. Two basic requirements have to be met for bioimaging. There needs to be a fast and sensitive method for detection, and the contrast agents or spectroscopic markers must meet all the requirements, i.e., biodegradability, biocompatibility, high specificity, sensitivity, and general applicability [100,101,102,103]. Bioimaging can track the development of abnormal processes, such as cancer development, hypoxia/hyperoxia, or necrosis. Functionalized CNMs can be used as probes in imaging techniques such as confocal fluorescence, surface-enhanced Raman scattering, coherent anti-Stokes Raman scattering, magnetic resonance, positron emission tomography, ultrasound, photoacoustic, and electron paramagnetic resonance imaging. All of these techniques have been extensively used for various bioapplications including bioimaging, biosensors, and drug delivery (Figure 3).

### 6.1. Application of Carbon Nanotubes

#### 6.1.1. Bioimaging

Magnetic resonance imaging (MRI) requires magnetic atoms in a contrast agent such as gadolinium (III) salts, iron oxide, or nitroxide radicals [106]. Functionalized SWCNTs filled with BiOCl/Bi${}_{2}$O${}_{3}$ [107] and radiocontrast agents such as I${}_{2}$ [108] and ${}^{125}$I${}^{-}$ [109] have been used for X-ray computed tomography. Na${}^{125}$I and ${}^{177}$LuCl${}_{3}$-filled SWCNTs [110], neutron-activated ${}^{153}$Sm sealed in SWCNT nanocapsules [111], and astatine (${}^{211}$AtCl)-filled SWCNTs [112] have been utilized for combined bioimaging and radiotherapy. The specific targeting of cancer cells was demonstrated by comparing SmCl${}_{3}$-filled amino-functionalized SWCNTs [113] and antibody-functionalized SWCNTs filled with SmCl${}_{3}$ and LuCl${}_{3}$ [114]. SWCNTs filled with PbO, BaI${}_{2}$ and Kr exohedrally functionalized with organelle-specific peptides were used as contrast agents for multiplexed X-ray fluorescence and Raman imaging, which allowed targeting and resolving subcellular structures (cell membranes, nuclei, and endoplasmatic reticulum) [115].

**Figure 4:**
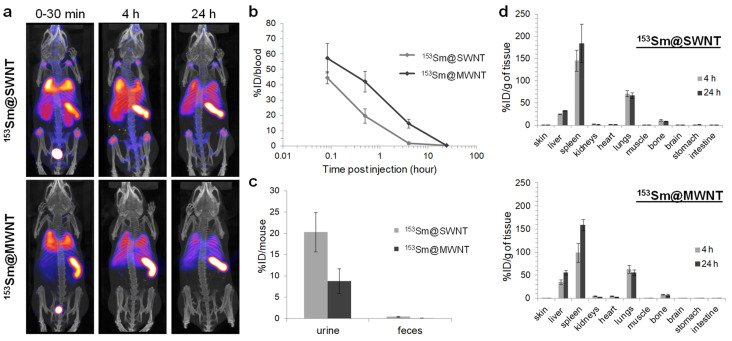
Whole-body SPECT/CT imaging and quantitative γ-counting that were used to study the pharmacokinetics and biodistribution of ${}^{153}$Sm@SWCNTs and ${}^{153}$Sm@MWCNTs up to 24 h. (**a**) SPECT/CT imaging, (**b**) blood circulation, (**c**) excretion profiles, and (**d**) tissue biodistribution profiles. Reprinted with permission from [111]. Copyright 2020, American Chemical Society.

In [111], a whole-body single-photon emission computed tomography/computed tomography (SPECT/CT) imaging and quantitative γ-counting were used to study the tissue biodistribution of ${}^{153}$Sm@SWCNTs and ${}^{153}$Sm@MWCNTs (Figure 4). The accumulation in the spleen, lung, and liver within 30 min was observed (Figure 4a). The blood circulation studies showed that the compounds were cleared from circulation at 4 h postinjection (Figure 4b). The excretion profiles revealed that roughly 20% for ${}^{153}$Sm@SWCNTs and 10% for the ${}^{153}$Sm@MWCNTs of the injected dose (ID) were detected in the urine, respectively (Figure 4c). Negligible radioactivity was observed in feces (<0.5% ID). The tissue biodistribution profiles showed that ${}^{153}$Sm@CNTs largely accumulated in the spleen, lung, and liver (Figure 4d).

#### 6.1.2. Drug Delivery

Nanomaterials have been extensively used for drug delivery applications [116,117,118,119]. If chemically functionalized CNMs, in particular, filled nanotubes, are used for drug delivery, they can greatly enhance the efficiency of the therapeutics. For the best possible benefit, the functionalized CNM-based drug delivery system needs to have good water solubility, good biocompatibility, and a high blood circulation time. The targeting and accumulation of therapeutics in diseased cells and tissues should be specific. The release of the therapeutic agent should be controllable and also targeted. At the same time, the toxic effect on healthy cells and tissues should be negligible, and after the therapy, the biodegradability of the functionalized CNMs is crucial [120]. Such targeted and efficient drug delivery systems have been realized lately with SWCNTs filled with DNA [121] and anticancer drugs such as hexamethylmelamine [122], irinotecan [123], indole [124], and cisplatin [125]. The release of drugs at the target can be triggered by electric stimulation, the pH of the medium, or temperature [120].

The development of drug delivery systems based on endohedrally and exohedrally functionalized CNMs is being actively researched. The research is focused on further developing and optimizing the methods of the chemical functionalization of CNMs. Increasing the degree of loaded therapeutic drugs and reducing the amount of nonloaded drugs are also important. There are methods to control the release of drugs, but better and more applicable methods are needed to achieve more efficient therapies. The solubility, biocompatibility, blood circulation time, biodegradability, and targeting of functionalized CNMs have to be improved, while their toxicity to normal cells has to be minimized. For these improvements, the underlying chemical and physical properties of the chemically functionalized CNMs need to be studied in detail. The therapeutic options can be further expanded by combining different agents, i.e., the codelivery of two and more drugs in functionalized CNMs. The development of a combined delivery of both biomedical contrast agents and drugs will in combination with a controllable release mechanism allow for an early diagnosis and local treatment of diseased tissues.

#### 6.1.3. Biosensing

Nanomaterials have also been actively studied as biosensing agents [126,127,128,129,130]. As mentioned earlier, in addition to unique optical properties, CNTs have a large surface area, which enables the attachment of a large number of functional groups, which makes them highly suitable for biosensing [131]. Biosensors based on CNT films are able to detect glucose, DNA, proteins, drugs, hormones, cancer cells, etc.

Glucose detection is one of the important applications of biosensors due to the need to control diabetes. Several promising CNT-glucose biosensors based on glucose oxidase were developed in 2004 [132,133]. Vilian et al. presented a glucose oxide biosensor based on multiwalled carbon nanotubes (MWCNTs), which was modified with the biopolymer L-arginine [134]. The copolymer based on photo-crosslinkable coumarin segments and carboxylic groups co-assembled with MWCNTs was also proposed as a sensitive and stable glucose detector [135]. Recently, ruthenium nanoparticle-functionalized MWCNTs served as a platform for the immobilization of a biotinylated glucose oxidase [136]. Furthermore, the gold/CNTs biosensor showed a low detection limit accompanied by high sensitivity [137].

It was found that single-stranded DNA absorbs strongly on CNTs in comparison to double-stranded DNA. This property can be used to build CNT–DNA biocomplexes for biosensing technologies to target various molecules [138]. The DNA sensor based on an SWCNT field effect transistor was presented as a high-density sensor [139]. The fluorescence polarization detection and MWCNT signal amplification in DNA biosensors were successfully implemented for DNA methyltransferase control and inhibition [140]. The gold nanoparticles/CNTs film DNA biosensor showed an enhanced stability and sensitivity [141].

MWNTs were also used in biosensors based on microsomal cytochrome P450 to detect drugs used in the treatment of breast cancer [142]. D${}^{\pm }$-galactose conjugated SWNTs were implemented as sensitive biosensors to detect a cancer marker—galactin-3 [143]. Folic-acid-functionalized polydopamine-coated CNTs were used for HeLa and HL60 cancer cell detection [144].

Overall, CNT-based biosensors have many advantages over commercially available sensors. Among them are the high sensitivity, due to their high surface-to-volume ratio and hollow tubular structure, fast response time, due to the fast electron transfer reactions, long lifespan, and high stability.

### 6.2. Applications of Graphene

#### 6.2.1. Bioimaging

In addition to CNTs, graphene and its derivatives are extensively explored for the improvement of the existing contrast agents, as well as developing completely new probes and agents in biomedical imaging. Different techniques are used to monitor the processes, not only in living cells, but also in tissues and the whole body [145].

As mentioned earlier, fictionalization is a key task of any type of imaging as it prevents the agglomeration of graphene in physiological buffers, which reduces the contrast and spatial resolution, in addition to increasing the biocompatibility. It is important to note that graphene itself is hydrophobic in nature [145], which makes it difficult to disperse in water and functionalize. In contrast, GO is hydrophilic due to the existence of oxygen, and therefore, it is water soluble [146]. The presence of a defect introduced by oxidation creates the chemically reactive sides in GOs [21], making them favorable for bio-imaging. Another important graphene derivative is rGO, produced by the thermal, chemical, or electrochemical treatment of GO [147]. It has a smaller number of oxygen groups than GO and a different ratio between carbon and oxygen; therefore, a higher level of functionalization can be achieved.

**Figure 5:**
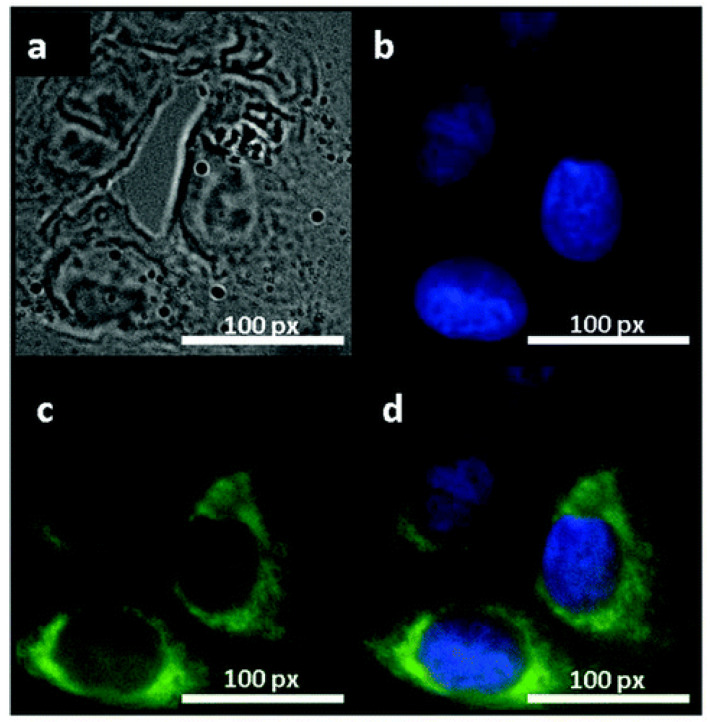
Fluorescent images of human breast cancer cells T47D after incubation with green GQDs for 4 h. (**a**) Phase contrast picture of T47D cells. (**b**) Individual nucleus stained blue with DAPI. (**c**) Agglomerated green GQDs surrounding each nucleus. (**d**) The overlay high-contrast image of the nucleolus stained with blue DAPI and GQD (green) staining. Reprinted with permission from [156]. Copyright 2012, American Chemical Society.

Unlike gapless graphene, GO shows a broad-band photoluminescence (PL) in the visible and nearinfrared (NIR) range excited by ultraviolet (UV). The usage of this intrinsic PL of nanographene oxide for NIR imaging of lymphoma cells was reported in [148]. GO has been extensively used for cancer cell imaging. It was also found that the fluorescence quantum yield (QY) of GO functionalized by PEG is relatively low, which limits its in vivo imaging. Therefore, further research has been focused on the improvement of fluorescence QY. In particular, organic fluorescent dyes to functionalize GO/rGO were actively studied, which allows performing not only in vitro, but also in vivo studies [149]. For example, the gelatine-grafted rGO labeled with a fluorescent dye was used for cellular imaging [75]. R6G red fluorescence was shown with a little background observed in the cytoplasm surrounding the nucleus, which was associated with the uptake of R6G@gelatin–GNS into the cytoplasm and the membrane of the cells. In addition to the improved biological contrast, Cy7-conjugated with GO–PEG has an enhanced permeability and retention effect with respect to cancerous tumors [88]. The PEG bridge between fluorescent dyes and GO and rGO prevents the quenching of fluorescence (FL) via charge and/or energy transfer processes. In addition, the fluorescence intensity of ischemic limbs is stronger than nonischemic limbs after injection of VEGF-loaded IR800-conjugated oxide, which suggests a different interaction of the contrast agent with hypoxic tissues [150]. In an equivalent manner, the contrast agents based on rGO conjugated by GQDs via a bridge of bovine serum albumin showed the enchantment of FL in intercellular imaging [151]. To overcome the quenching of the FL in dye–graphene systems, sinoporphyrin-sodium-loaded GO was also used, which opens a new direction in photodynamic therapy with optical imaging [152]. The FL intensities of GO–PEG–DVDMS composite were 3–8-times higher than that of the pure GO with a different composition of components. The further improvement of the biological contrast was achieved by using graphene QDs on their own. The water-soluble, stable, and low-cytotoxicity graphene QDs with strong PL were employed to visualize stem, MG-63, HeLa, T47D, and MCF-7 cells [153,154,155,156,157]. Figure 5a–d shows the images of T47D cells treated with green GQDs for a 4 h incubation time. The obtained images clearly visualize the phase contrast image of T47D cells, the nucleus stained blue with DAPI, blue-fluorescent DNA stain, agglomerated high-contrast fluorescent image of green GQDs around each nucleus, and the overlay image of a cell with phase contrast, DAPI, and green GQDs. These obtained images show that GQDs can be used in high-contrast bioimaging and other biomedical applications. The multispectral contrast agent based on GQD showed an FL QY of 54.5%, which is highly suitable for both in vivo an in vitro imaging [158].

The strong D and G Raman modes commonly observed in graphene can be further enhanced by noble metal nanoparticles’ deposition. The gold and silver nanoparticles on the graphene surface result in surface-enhanced Raman scattering (SERS). GO decorated by gold nanoparticles used for Raman imaging of HeLa229 cells showed a stronger and up to 192 better contrasted images than for GO [159]. The silver nanoparticles and GO hybrids were confirmed as good SERS contrast agents for cancer cells [160]. Importantly, the selectivity of such composites to the concentration in certain cells was confirmed: GO–Ag nanoparticles (NPs) showed a stronger Raman signal on the plasma membrane of HeLa cells, while no signal was observed on cancer-free A549 cells.

The two-photon fluorescence of graphene-based materials results in creating highly efficient contrast agents for deep-sited organs due to the infrared two-photon excitation located in the NIR and middle-infrared (MIR) ranges in biological transparency windows. As an example, the growth of a whole zebrafish embryo treated by GO was dynamically imaged by using two-photon fluorescence [161]. The nitrogen-doped QDs showed the two-photon absorption suitable for deep-tissue imaging [162]. Importantly, S6 RNA aptamer-conjugated GO-based two-photon luminescence showed selective imaging of breast cancer cells in second biological transparency windows [163]. The studies showed the penetration depth up to 1800 µm achieved during the two-photon fluorescence emission in contrast to 400 µm during the one-photon emission [162].

**Figure 6:**
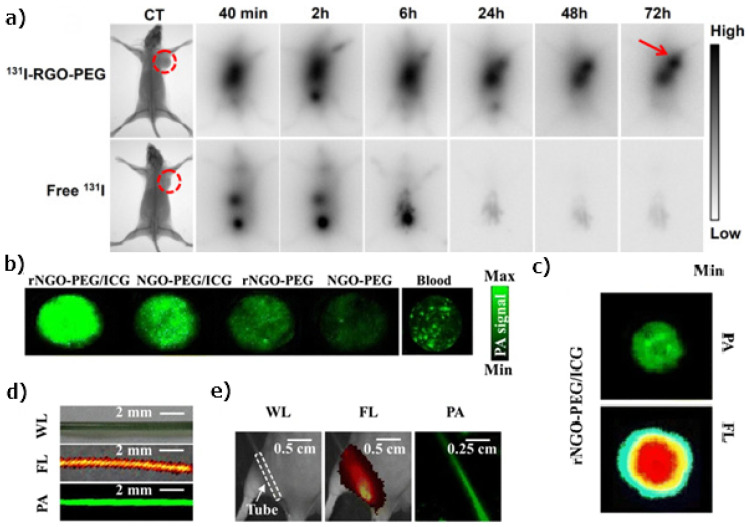
(**a**) In vivo behaviors of ${}^{131}$I–rGO–PEG. Gamma imaging of mice bearing 4T1 tumors after i.v. injection of ${}^{131}$I–rGO–PEG and free ${}^{131}$I at the same radioactivity dose. ${}^{131}$I–rGO–PEG can passively accumulate in the tumor via the enhanced permeability and retention (EPR) effect, while ${}^{131}$I can only be rapidly excreted from the body. Tumors are highlighted by circles in X-ray imaging and arrows in gamma imaging [164]. Copyright 2015, with permission from Elsevier. (**b**) Photoacoustic images of NGO–PEG, rNGO–PEG, NGO–PEG/ICG, and rNGO–PEG/ICG with the same GO concentration and whole blood. (**c**) Photoacoustic and FL images of rNGO–PEG/ICG covered with 5 mm-thick agarose gel containing 0.5% intralipid. (**d**) White light (WL), FL, and PA MAP images of a PE tube filled with rNGO–PEG/ICG. (**e**) White light, FL, and PA MAP images of a mouse with the rNGO–PEG/ICG-filled PE tube implanted subcutaneously at the dorsal aspect of the leg. The white dashed box indicates the location of the tube [169].

To overcome the penetration depth limit and quenching effect, typical for optical methods, radionuclide-based imaging methods are commonly used. GO edges and defects are used to attach a number of labels such as ${}^{125}$I, ${}^{64}$Cu, ${}^{66}$Ga, and ${}^{198/199}$Au, which showed a tumor-targeting behavior [164,165,166,167]. In vivo studies were performed on mice with U87MG tumors targeted by ${}^{64}$Cu-labeled GO. The design and synthesis of a PEG-coated rGO labeled with ${}^{131}$I was described in [164]. The targeting properties of ${}^{131}$I–rGO–PEG can be seen in Figure 6a: ${}^{131}$I–rGO–PEG can passively accumulate in the tumor, while ${}^{131}$I can only be rapidly excreted from the body. A radiolabel GO–aminopropylsilyl derivative with ${}^{198/199}$Au nanoparticles to target and visualize fibrosarcoma tumors allowed a fast tumor visualization with a fast removal from the body via the kidneys [168].

In contrast to the above-described methods, MRI is a noninvasive method with a high spatial resolution. GO is a diamagnetic material and cannot be considered as a contrast agent for magnetic resonance imaging. Only the manipulation with defects and oxygen groups of GO allow finding magnetism in this structure. A positive T1 MRI GO contrast agent (GO–DTPA–Gd/DOX) based on GO–gadolinium (Gd) complexes offered a dual-modality: MRI/fluorescence imaging and drug delivery [170]. In particular, the authors showed that such a contrast agent can be used for cell imaging and tracking. Li et al. fabricated a GO/BSA–Gd${}_{2}$O${}_{3}$/AS1411–DO complex with BSA–Gd${}_{2}$O${}_{3}$ NPs intended to be used as an MRI contrast agent, showing also the targeting and growth of human renal carcinoma 786–0 cells [171]. The contrast agent consisted of GO with acid as an anticancer agent with Gd(III) nitrate hexahydrate combined with AuNPs showing not only perfect contrast properties, but also HepG${}_{2}$ cancer cell oppression.

Finally, to overcome the problem with a penetration depth, photoacoustic imaging (PIA) based on graphene can be used. Graphene-based nanomaterials have been widely investigated due to the high NIR absorption and conversion to acoustic waves. The rGO and iron oxide nanoparticles were used as a contrast agent in photoacoustic tomography [172]. The indocyanine-green-conjugated rGO nanocomposite was suggested for the multimodality use of PAI and FL imaging [169]. This composite exhibits a low toxicity and high PA signal in combination with tumor-targeting both in vivo and in vitro. Figure 6b shows the photoacoustic images of NGO–PEG, rNGO–PEG, NGO–PEG/ICG, and rNGO–PEG/ICG with the same GO concentration. It can be seen that the photoacoustic image presents a much sharper boundary of the target (Figure 6c). The white light (WL), FL, and PAI images of a polyethylene (PE) tube filled with rNGO–PEG/ICG before (Figure 6d) and after (Figure 6e) being implanted into the leg of a living mouse are shown.

#### 6.2.2. Drug Delivery

The areas of graphene in GO contain free π electrons and, therefore, are hydrophobic and capable of drug loading and noncovalent surface modification by π–π stacking and hydrophobic interaction. Graphene and GO became important drug delivery agents due to their loading capability. They are also considered as antimicrobial agents, which can enhance the efficiency of the delivered antimicrobial drugs [173]. In addition, under NIR laser irradiation, graphene shows controlled cytotoxic effects [174]. In addition, pH modulation is one of the commonly used strategies in cancer targeting by GO and drug release from the GO’s surface [175]. Most of the drug delivery applications of graphene are focused on cancer treatment. However, several important applications of noncancer therapeutics have also been developed.

The drug delivery capability of GO was reported for the first time by Liu and coworkers [176]. In this article, GO–PEG was loaded with SN38. This complex exhibited good water solubility and high cytotoxicity in HCT-116 cells. The nanoscale graphene oxide (NGO) was also used for the loading and targeted delivery of anticancer drugs [177]. The pH-dependent drug delivery for cancer treatment using GO was shown in some research. In particular, doxorubicin (DOX) immobilized on the surface of GO along with the CD 20+ antigen and B-lymphocyte antigen using PEG showed the selective release of DOX inside only cancer cells due to the acidic environment [178]. Synthesized PEG–GO to deliver water-soluble anticancer drug SN38 to colon cancer cells was performed [176]. The PNIPAM–GS–CPT nanocomposite showed a strong potency towards in vitro cancer cell killing [175]. The gold nanoclusters and rGO loaded with DOX were successfully used for targeted delivery to cancer (hepatocarcinoma) cells [151].

Graphene–carbon nanotube–iron oxide binds the anticancer drug 5-FU, leading to a high loading capability and pH-dependent drug release [179]. A multimodality therapeutic agent-based SiO${}_{2}$-coated quantum dot conjugated folic acid graphene loaded with DOX showed cancer targeting. drug delivery, and the ability to be used for fluorescence imaging. This allows not only the delivery of the drug, but also the tracking of the delivery process and the monitoring of cellular uptake [180]. Another way to control drug delivery is the so-called triggered drug delivery, which provides control over the drug dose depending on the physiological response [181]. Another control of anticancer effects via light-emitting diode (LED) illumination was studied by the codelivery of DOX and Ce6 by GO nanosheets [182].

The efficient treatment of hepatitis in mice was performed by graphene QDs via the preferential accumulation of the liver and the reduction of Con A-mediated liver damage [183]. Primidone loaded on GO has an ultrahigh loading capability and a high cell penetration performance, which results in the quick delivery of the drug to the brain tissue in mice. Moreover, the pirfenidone–GO complex is not toxic at the low concentrations that are used for treatment [184]. In addition, this indicates the brain targeting properties.

Importantly, GQDs show an inhibitory activity related to drug-resistant viruses and bacteria [185]. GO also shows strong bacteriostatic and bactericidal properties in addition to the inhibition of bacterial growth [186]. Both in vitro and in vivo studies showed that GO reduces Klebsiella pneumonia growth and spread. The GO flakes most likely cause damage to the cellular membrane and result in the leakage and release of the cell content, and therefore cell damage [187]. Graphene and GO coated with copper (Cu) and gallium (Ga) inhibit bacterial growth [188]. However, contaminations and graphene doping with nitrogen, sulfur, boron, or phosphorus atoms in GO may have a significant impact on its antimicrobial efficacy [189]. The synergetic antimicrobial activity of common antimicrobial agents and GO was also shown via enhancement of drug resistance due to the presence of GO [190,191]. In addition, such components together show an enhanced antimicrobial effect higher than the sum of the effects from the individual agents.

Graphene was shown as an effective intracellular and protein delivery agent [192,193]. The delivery of DNA that encodes a therapeutic gene to replace mutated genes using graphene has been investigated [194]. Graphene has been found to be an ideal candidate for the nuclear delivery of small interfering RNA (siRNA) and micro-RNA (miRNA), for therapeutic purposes [195]. Graphene can also deliver very small peptides intercellularly, as demonstrated in [12,196].

#### 6.2.3. Biosensing

Graphene and its derivatives have been employed in the design of different biosensors of various types because of their large surface area, bright fluorescence, high electrical conductivity, and high electron transfer rate. Graphene has been extensively explored for glucose sensors [192]. Several studies showed the direct electron transfer to graphene, which allows the glucose oxidase to be detected by electrochemical sensing [197]. A higher sensitivity of glucose detection was achieved by the deposition of nanocrystals [198]. Nicotinamide adenine dinucleotide’s (NADH) electrochemical behavior was studied on rGO sheets [199]. Moreover, a multitask detection was also shown: the low potential electrochemical detection of NADH and ethanol was performed using ionic-liquid-functionalized graphene [200]. For the treatment of genetic diseases, DNA detection was achieved using GO [201], epitaxial graphene [202], and graphene QDs [203]. The hemoglobin content in the human body using the chitosan–graphene complex as an electrode showed a well-defined difference in the voltammogram in comparison to a chitosan electrode [204]. Furthermore, the selectivity and sensitivity to the low-concentration detection of Bovine hemoglobin were achieved using molecular graphene [205]. Dey et al. developed a multitask detection of separately H${}_{2}$O${}_{2}$ and cholesterol on the surface of platinum–graphene complexes [206].

**Figure 7:**
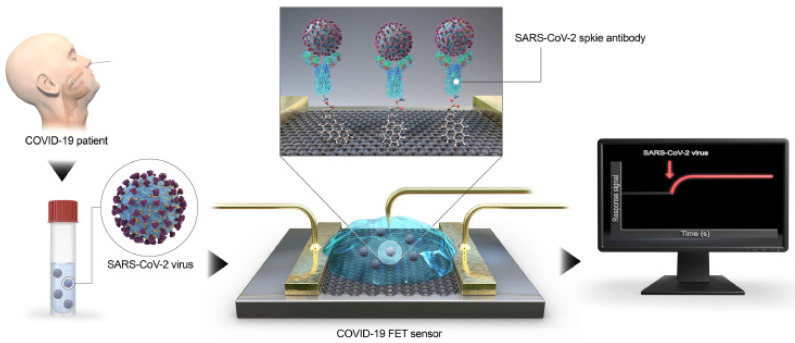
SARS-CoV-2 detection scheme using GFETError. Reprinted with permission from [224]. Copyright 2020, American Chemical Society.

Graphene was also employed as a sensitive substrate for fluorescence detection systems [207]. It was successfully used for sugar ligands’ detection and glycerin on cancer cells [208]. The quenching of the fluorescence of certain DNA on GO can be used for selective biosensing [209]. Chen et al. demonstrated a GO-based fluorescent sensor for detecting dopamine [210]. Nobel nanoclusters such as Ag and Au also show promise for greatly enhanced detection limits and sensitivity of fluorescence-based biosensors. Surface plasmon resonance (SPR) is widely used in biological investigations of molecules. A Ag nanocluster–GO hybrid system was developed as a fluorescent DNA sensor for cancer-related enzymes [211]. Au–GO-based sensors enabled the sensitive detection of thrombin [212] and lysozyme in serum [213]. The dual amplification strategy based on a DNA–GO–AuNP-functionalized sensor and the upper layer was used to perform the sensitive detection of miRNA and adenosine [214]. A graphene-based SERS immunoassay is usually applied to the quantitative detection of different kinds of proteins such as IgG [215], cTnI [216], and β-amyloid [217]. A universal fluorescence biosensor based on graphene QDs possesses unique size-dependent fluorescence properties. It was suitable to detect protein kinase [218], glucose [219], and pesticides [220]. As a zero-band-gap semimetal, graphene is an ideal candidate for the fabrication of field effect transistor (FET)-based biosensors. FETs based on graphene and GO are ideal for the detection of charged molecules, such as DNA, even in a single-stranded form [221,222]. The increase of the biosensor sensitivity was also achieved by the deposition of Au and Ag nanoparticles [223]. Furthermore, it has been discussed how to use graphene-based FET for viruses’ analysis, in particular COVID-19 [224,225]. The fabricated GFET (Figure 7) was functionalized with the SARS-CoV-2 spike antibody for the detection of SARS-CoV-2 [224].

### 6.3. Application of Graphene Nanoribbons

#### 6.3.1. Bioimaging

Unlike graphene, the lateral quantum confinement imposed by their finite width opens a sizable electronic band gap, which makes GNRs and its derivatives highly suitable for optical imaging [226]. The first in vivo fluorescence images using nanographene sheets functionalized by PEG was shown in [227]. PEG biocompatible GO with different shapes was also prepared as a drug carrier [228]. SN38 was loaded onto PEG–nano-GO sheets using π–π interactions [148]. The GNRs can emit light in a solid state, here optical absorption and fluorescence along with photoluminescence spectra, which can be used as a contrast agent in photoluminescence imaging [229].

With diffraction-limited fluorescence microscopy and super-resolution microscopy imaging techniques, the structure of single-dye-functionalized GNRs coated on transparent and opaque insulating substrates from dilute solution can be resolved. oGNRs can also be reduced to rGNRs, a modification that also improves the NIR absorption and photothermal capacity, but reduces the solubility [228]. Gd${}^{3+}$-ion-conjugated carboxyphenylated rGNRs in aqueous solution are one of the promising contrast agents for MIR in comparison to the individual counterparts. GNRs have been also reported as contrast agents for photoacoustic and thermoacoustic imaging and tomography [230].

#### 6.3.2. Drug Delivery

**Figure 8:**
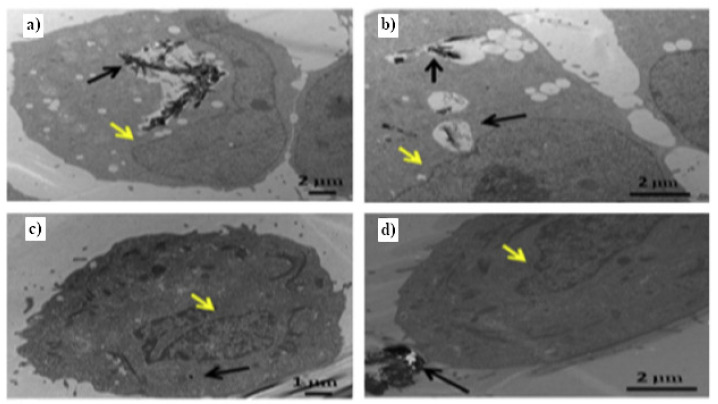
(**a**) The uptake of GONR–PEG–DSPE into U251 cells in large vesicular structures (black arrow); (**b**) the uptake of small aggregates of GONR–PEG–DSPE into U251 cells (black arrow); (**c**) no or minimal uptake of GONR–PEG–DSPE into MCF-7 cells (black arrow); (**d**) large aggregates outside of the MCF-7 cells (black arrow) [231]. Copyright 2015, with permission from Elsevier.

In comparison to the rest of the carbon-based materials, the usage of GNRs arises from the combination of the abundant oxygen-containing functionalities and the large loading surface area. oGNRs and rGNRs are used in cancer therapy and can be functionalized by the same procedures described for GO. Therefore, GNRs have been proposed as a unique drug delivery agent for tumor therapy. The phospholipid–polymer-conjugate-functionalized oGNRs, oGNR–PEG–DSPE, can be considered a good vessel for delivering lucanthone (Luc) to glioblastoma multiform (GBM) tumors [231]. Higher uptake and cytotoxicity have been shown for glioblastoma multiforme cells (Figure 8). PEG–oGNRs can be used as an efficient delivery vehicle for anticancer drugs by facilitating intracellular drug delivery, as shown in [232]. In addition, DSPE–PEG–NH${}_{2}$-coated rGONR induces cytotoxic and genotoxic effects on glioblastoma cells [228]. DSPE–PEG–RGD-functionalized rGONR achieves a higher internalization in glioblastoma cells than its equivalent functionalized with DSPE–PEG–RAD, leading to an enhanced photothermal effect [228].

**Figure 9:**
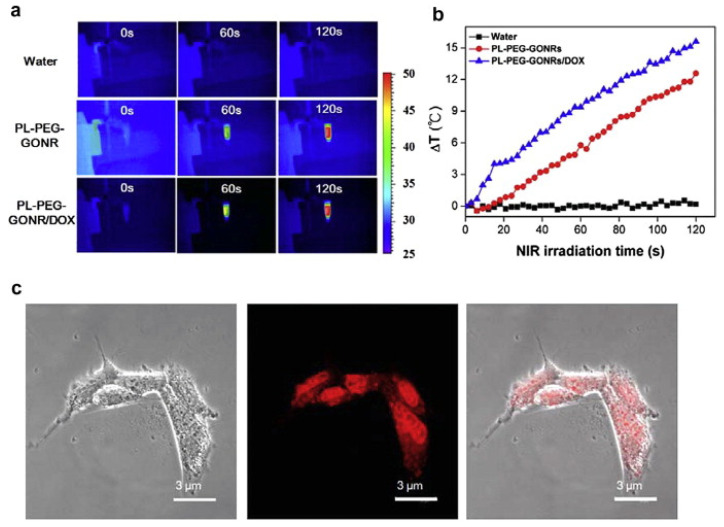
(**a**) NIR thermal images of water, PL–PEG–GONR, and DOX-loaded PL–PEG–GONR; (**b**) graph showing 120 s NIR irradiation with increasing temperature; (**c**) bright field (**left**), fluorescence (**middle**) and merged micrographs (**right**) of U87 cells after treatment with PL–PEG–GONRs/DOX for 6 h observed by confocal laser scanning microscopy [232]. Copyright 2014, with permission from Elsevier.

Polyethylenimine-grafted graphene nanoribbons (PEI-g–GNRs) were proposed as an effective gene vector drug carrier with the targeting of HeLa cells. Moreover, a novel oxidized graphene nanoribbon-based platform (oGNR) for gene delivery of double-stranded DNA into mammalian cells was performed. It exhibited efficient DNA loading of small double-stranded (ds) DNA fragments [233]. Some studies showed a great cellular response and drug delivery ability of O–GNR-based nanoparticles that was different from other carbon materials [231,234]. It was also proposed for nuclear gene delivery [235]. The excellent NIR absorbance of PL–PEG–GONR hybrids shown in Figure 9a allows for effective photoheating to high temperatures (Figure 9b), which can be used to kill tumor cells, thus rendering PL–PEG–GONRs/DOX as a candidate for photothermal therapy [232]. Cellular uptake experiments using GONR showed that the uptake of PL–PEG–GONRs/DOX was observed after 6 h and that most of the PL–PEG–GONRs/DOX appeared to be taken up by endocytosis into the cytoplasm, then the released DOX entered the nuclei (Figure 9c). The size and shape of GNRs, similar to graphene flakes, result in antibacterial and antimicrobial properties due to the oxygen-containing functionalities, which produce stresses in microorganisms, which enhances drug efficiency [236]. Moreover, the atomically sharp edges of GNRs more actively damage the cell membrane, resulting in the leakage of electrolytes and RNA and consequent cell death [237].

#### 6.3.3. Biosensing

Nonoxidized GNRs may be more appropriate for use as biosensors in which the higher surface area is a key factor for assembling polymers and DNA [238]. In addition to graphene, GONRs and graphene sheets/graphene nanoribbons/nickel nanoparticles complexes can be used as substrate electrodes for glucose and hydrogen peroxide sensing [239,240]. Ultrasensitive targeting of DNA using nanoparticle-functionalized GNRs was shown, as well as detecting by GNR-based FET [241,242]. Reduced graphene nanoribbons (rGNRs) were found to be sensitive to ascorbic acid, dopamine, NADH, and DNA bases such as guanine [243]. GNRs functionalized by Ag@Pt demonstrated the ability to sense immunoglobulin, which is important for a number of diseases such as atherosclerosis, influenza A virus, abnormal prothrombin, carcinoembryonic antigens, etc. [244]. Ag-functionalized graphene also showed the ability to detect galactin-3 biomarkers for heart failure and neurotransmitters from serum samples [245]. GNRs are able to detect microalbuminuria (mAlb) for the diagnosis and treatment of nephritis and hypoproteinemia [246]. Nitrogen-doped GNRs with an amine aptamer have been used for the detection of aflatoxin B1 [247]. The size-dependent properties improved the sensitivity and affinity of nGNR to proteins. The hybrid material consisting of porous GNRs and AuNPs immobilized with anti-alpha fetoprotein (anti-AFP) deposited on a glassy carbon electrode (GCE) (anti-AFP–AuNPs–PGNR–GCE) exhibited high electrocatalytic activity towards the alpha-fetoprotein molecule, which can be used in the treatment of numerous diseases [248]. The sensing of cholesterol in the near-IR range based on the CdTe quantum dots (QDs) arranged on MWCNTs at reduced GONRs has been developed [249]. Adenosine triphosphate (ATP) molecules can be effectively detected by FET-based sensors [250].

## 7. Toxicity Studies of Carbon Nanomaterials

Nanomaterials have been used in numerous biomedical applications. However, considerable attention needs to be paid to the human and environmental risks of these materials. For this reason, it is important to evaluate the level and degree of toxicity, biocompatibility, and biodegradation of nanomaterials [251,252,253]. So far, CNMs still exhibit a toxic effect on biological systems.

In comparison to the rest of the carbon-based nanomaterials, CNTs containing catalyst metal ions that are incorporated inside of the CNTs are toxic to cells [254]. These metals include Co, Fe, Ni, and Mo, all of which have documented toxic effects. Purified CNTs, which contain a lower metal content, have shown less pronounced or nontoxic effects [255]. In addition, it was shown that the cellular uptake and damage highly depend on the CNTs’ length and diameter [256]. In fact, higher-diameter and similar-length CNTs exhibit greater toxicity [257]. Moreover, the level of CNT toxicity depends on the agglomeration state of SWCNTs. Dispersed SWCNTs showed an increased aspect ratio relative to the resulting aggregates. Phagocytic cells were able to eliminate individual SWCNTs and reduce toxicity [258]. Some of the surfactants used to disperse CNTs also show a high level of toxicity. Among them, Pluronic F127 showed better viability with a low level of toxicity. In addition, the uncoated CNTs were found to be more cytotoxic compared to Pluronic-F127-coated ones [259]. The toxicity of CNTs also depends on the functional groups attached to the CNT walls. For example, it was found that CNT–COOH has higher toxicity in comparison to nonfunctionalized SWCNTs with respect to the HUVEC cell line. Several studies have identified oxidative stress as a common mechanism of CNT-induced cell toxicity [260]. In particular, concentration-dependent cytotoxicity in cultured HEK293 cells was associated with increased oxidative stress by CNTs [261]. It was also shown that the accumulation of SWCNTs in lysosomes induces reactive oxygen species generation, which in turn triggers lysosomal membrane damage, causing apoptosis and necrosis [262].

The shape, surface chemistry, purity, morphology, surface charge, functionalization, dispersion, and number of layers play important roles in the toxicity of graphene [263]. Traditionally, prepared GO often contains high levels of Mn${}^{2+}$ and Fe${}^{2+}$, which results in cytotoxicity and DNA fracturing [264]. The toxicity of graphene highly depends on its size and hydrophobicity. Graphene and its derivative flakes can enter the cytoplasm because of their small size and sharp edges. This causes damage to the cell membrane, leading to leakage of the cytoplasmic content. In fact, small hydrophobic graphene flakes were found to be less toxic than larger particles. In particular, large graphene flakes (780 nm) generated more reactive oxygen species than smaller ones (160, 430 nm) [265]. In comparison to rGO, GO showed higher DNA toxicity than graphene due to oxidative stress. Furthermore, an agglomeration of graphene flakes inside the cell leads to the disruption of the cell interior, membrane deformation, and intercellular stresses with consequent cell death. It was shown that GONR is toxic to human A549 adenocarcinoma cells at a concentration between 3–400 μg/mL [266].

Similar to CNTs, the toxicity of graphene highly depends on the functionalization. It has been shown that carboxyl functionalization can significantly decrease graphene’s toxicity [267]. However, the hydrophilic functionalization of graphene results in the formation of reactive oxygen species in mammalian cells and enhanced cytotoxicity [268]. In addition to the toxicity of graphene to cell lines, some studies have revealed the toxicity of graphene and its derivatives to human erythrocytes. Liao and coworkers found severe hemolysis due to the electrostatic interaction between the lipid bilayer of the erythrocyte membrane and the surface of graphene, which disrupted the cell membrane [268]. The injection of graphene and its derivatives into the body resulted in their accumulation in the lung and kidney for expulsion due to the interaction with immune cells. Higher doses of graphene flakes can significantly affect organs’ functionality, resulting in their failure and even death [269]. Due to its small size, high surface area, and surface charge, GO also causes severe DNA damage [270,271].

## 8. Conclusions

In this review, we comprehensively described the latest achievements in the chemical functionalization routes and applications of CNTs, graphene, and GNRs. We discussed noncovalent and covalent exohedral modification approaches, as well as endohedral functionalization methods. The methods to improve the functionalities of CNMs were highlighted. In addition, the applications of functionalized CNMs in bioimaging, drug delivery, and biosensors were extensively reviewed. The study of the biocompatibility of GNMs is still limited. Therefore, we summarized the toxicity studies of CNMs. Overall, this review tracked several challenges, for example the appropriate functionalization and biocompatibility, that need to be resolved to improve the bio-applications and to bring them from the research laboratory to the clinic. We believe that our review will also help to guide future research.

## 9. Outlook

The further directions for the development of the field include:

1. The development of methods of the surface functionalization of CNMs to improve the solubility, biocompatibility, biodegradability, and targeting of functionalized CNMs and reduce their toxicity;
2. The development of methods of the loading of CNMs with therapeutic drugs to increase the degree of loading and reduce the amount of nonloaded drugs;
3. The comparison of the efficiency of the methods of the external and internal loading of CNTs with therapeutic drugs;
4. The revealing of the correlation among the structural parameters of CNMs (i.e., diameter and length of CNTs, the size of GQDs), surface functionalization, and their accumulation in tissues and cells;
5. The development of the methods of controlling the release of drugs from loaded CNMs (pH of the medium, temperature, electric stimulation);
6. The development of the methods of the loading of CNMs with contrast agents for bioimaging to increase the degree of loading and reduce the amount of nonloaded contrast agents;
7. The controllable modification of the physicochemical properties of CNMs loaded with contrast agents to improve the sensitivity of imaging and detection, to provide a high spatial resolution and imaging of deeper tissues;
8. The development of the methods of the combined delivery of both biomedical contrast agents and therapeutic drugs using CNMs.

## Data Availability

Not applicable.

## Abbreviations

The following abbreviations are used in this manuscript:

ATP	Adenosine triphosphate
CNMs	Carbon nanomaterials
CNTs	Carbon nanotubes
CT	Computerized tomography
DNA	Deoxyribonucleic acid
dsDNA	Double-stranded DNA
FET	Field effect transistor
FL	Fluorescence
GBM	Glioblastoma multiform
GCE	Glassy carbon electrode
GNRs	Graphene nanoribbons
GO	Graphene oxide
GQDs	Graphene quantum dots
LED	Light-emitting diode
Luc	Lucanthone
miRNA	Micro-RNA
MIR	Middle-infrared
MRI	Magnetic resonance imaging
MWCNT	Multiwalled carbon nanotube
NADH	Nicotinamide adenine dinucleotide
NGO	Nanoscale graphene oxide
NIR	Nearinfrared
oGNRs	Oxidized graphene nanoribbons
PAH	Polycyclic aromatic hydrocarbon
PAI	Photoacoustic imaging
PE	Polyethylene
PEG	Polyethylene glycol
PEI	Polyethylenimine
PVP	Polyvinylpyrrolidone
QY	Quantum yield
rGNRs	Reduced graphene nanoribbons
rGO	Reduced graphene oxide
RNA	Ribonucleic acid
SERS	Surface-enhanced Raman scattering
siRNA	Small interfering RNA
SPECT	Single-photon emission computed tomography
SPR	Surface plasmon resonance
SWCNT	Single-walled carbon nanotube
WL	White light
